# The Crystal Structure of *Bacillus cereus* HblL_1_

**DOI:** 10.3390/toxins13040253

**Published:** 2021-03-31

**Authors:** Harley L. Worthy, Lainey J. Williamson, Husam Sabah Auhim, Stephen H. Leppla, Inka Sastalla, D. Dafydd Jones, Pierre J. Rizkallah, Colin Berry

**Affiliations:** 1 School of Biosciences, Cardiff University, Park Place, Cardiff CF10 3AX, UK; H.Worthy@exeter.ac.uk (H.L.W.); WilliamsonLJ@cardiff.ac.uk (L.J.W.); almaslookhihs@gmail.com (H.S.A.); jonesdd@Cardiff.ac.uk (D.D.J.); 2 The Henry Wellcome Building for Biocatalysis, Exeter University, Stocker Road, Exeter EX4 4QD, UK; 3 Department of Biology, College of Science, University of Baghdad, Baghdad, Iraq; 4 Laboratory of Parasitic Diseases, National Institute of Allergy and Infectious Diseases, National Institutes of Health, Bethesda, MD 20892, USA; sleppla@niaid.nih.gov (S.H.L.); inka.sastalla@nih.gov (I.S.); 5 Scientific Review Program, Division of Extramural Activities, NIAID, NIH, Rockville, MD 20892, USA; 6 School of Medicine, Cardiff University, Heath Campus, Cardiff CF14 4XN, UK; RizkallahP@cardiff.ac.uk

**Keywords:** tripartite toxin, hemolytic toxin, haemolytic toxin, *Bacillus cereus*

## Abstract

The Hbl toxin is a three-component haemolytic complex produced by *Bacillus cereus sensu lato* strains and implicated as a cause of diarrhoea in *B. cereus* food poisoning. While the structure of the HblB component of this toxin is known, the structures of the other components are unresolved. Here, we describe the expression of the recombinant HblL_1_ component and the elucidation of its structure to 1.36 Å. Like HblB, it is a member of the alpha-helical pore-forming toxin family. In comparison to other members of this group, it has an extended hydrophobic beta tongue region that may be involved in pore formation. Molecular docking was used to predict possible interactions between HblL_1_ and HblB, and suggests a head to tail dimer might form, burying the HblL_1_ beta tongue region.

## 1. Introduction

The tripartite haemolysin BL, Hbl toxin has been proposed as a causative agent of *Bacillus cereus* diarrhoeal syndrome [1]. It may be produced by bacteria in the *Bacillus cereus sensu lato* group including strains of *B. cereus sensu stricto* and some *Bacillus thuringiensis* strains [2,3]. There is considerable variation in the amino acid sequences, and strains may carry more than one *Hbl* operon [4,5,6]. These proteins are associated with dermonecrotic vascular permeability and both the diarrhoeal food poisoning and non-gastrointestinal infections caused by *B. cereus* [7]. Sequential CRISPR-based screens identify LITAF and CDIP1 as the *B. cereus* haemolysin BL toxin host receptors [8]. Insertion of Hbl components to form the lytic pore results in potassium efflux from the cell and this triggers activation of the NLRP3 inflammasome, interleukin release and pyroptosis, inducing inflammasome-mediated mortality [9].

The toxin is composed of three components HblB, HblL_1_ and HblL_2_, all of which are essential for activity with no individual or pair wise activity [10]. In haemolysis assays on blood agar plates Hbl produces a characteristic discontinuous pattern of lysis [7], which appears to be due to the inhibitory effect of excess concentrations of HblB and HblL_1_ components (>1.3 nM) [11]. Although it has been reported that all components are able to bind to erythrocytes independently [11], more recent work strongly suggests that only the HblB component is able to bind and, thereby, to carry out a rate limiting priming step to allow lysis by the L components [4]. Optimum ratios of the three components have been analysed and a sequential binding of B-L1-L2 components has been proposed [4,12].

A crystal structure of the HblB component was published in 2008 [13] and all components are predicted to share a similar fold, characteristic of the alpha pore-forming toxins (α‑PFTs), also related to the Nhe toxins of *B. cereus* (another 3-part toxin), the HlyE toxin from *Escherichia coli* and the App6 pesticidal protein from *B. thuringiensis* [14] (known as Cry6 prior to a recent nomenclature revision [15]). However, the structure of other Hbl components have not previously been reported. Here, we describe the structure of the HblL_1_ protein at 1.36 Å resolution, compare the structure to other α‑PFTs and model possible interactions between HblL_1_ and HblB.

## 2. Results and Discussion

### 2.1. Structure Description

Diffraction extended to 1.36 Å resolution, with good statistics in space group P 2_1_ 2_1_ 2_1_ (Table 1). The electron density map showed continuous density for residues 41 to 404 (accession EEM59260.1) of the protein sequence. The nascent protein is produced with a signal peptide that is cleaved and mass spectrometry shows a major peak at 41,463.29 and a minor peak at 41,681.28 corresponding to cleavage after alanine 30 and alanine 28 (theoretical 41,462.78 and 41,681.04, respectively). The remaining 10 or 12 N-terminal residues of the protein along with the native C‑terminal residues Q405-E406 and the C-terminal His tag were disordered and, therefore, not observed in the map.

**Figure 1:**
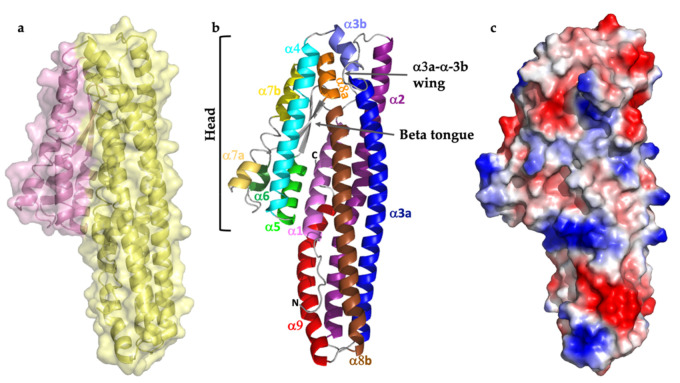
Structure of HblL_1_. (**a**) Domain structure with domain 1 in yellow and domain 2 in pink; (**b**) annotated with the 9 alpha helices labelled (with discontinuous helices labelled “a” and “b”) and key features shown, (**c**) surface charge distribution.

The overall fold comprises two almost parallel helical domains (Figure 1a). Domain 1 is a bundle of five helices covering residues G41 to Q210 and R315 to K404. Domain 2 is a pair of two-helix bundles with an interleaving two strand β-sheet, covering the middle stretch of the sequence from residues A211 to D314 (Figure 1). The interface between the two domains involves 45 residues from each, making 15 hydrogen bonds and 1 salt bridge. Interface analysis by PISA estimates the interface area at 1848 Å^2^ and the energy required for dissociation at 27.6 kcal mol^−1^. The points where the chain transitions between the two domains (residues 209–212 and 313–316) show some of the highest b factor scores in the structure and may represent hinge regions through which the two domains could move apart, although in the present structure they are in a closed conformation. The presence of a putative hinge region and potential flexibility is consistent with the large rearrangements seen in the structurally related HlyE (also known as ClyA or SheA), on pore formation [16,17].

Crystal packing of the monomers (Figure S1) does not show specific interactions between symmetry related copies, with the largest interface area at ~440 Å^2^. No super structure or macro cluster of HblL_1_ is likely to exist in solution as a biological entity.

### 2.2. Comparison with Related Structures

**Figure 2:**
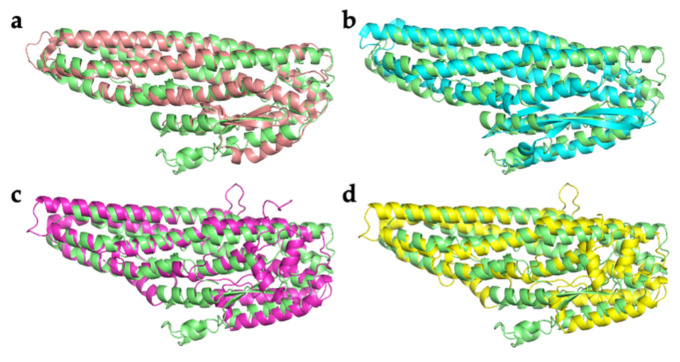
Comparison of HblL_1_ with structural homologs. (**a**) HblB (2NRJ, pink); (**b**) NheA (4K1P, cyan); (**c**) App6Aa2, trypsin-activated (5KUC, magenta); (**d**) App6Aa2 (5KUD, yellow). HblL_1_ is shown in green in all comparisons.

Alignment of HblL_1_ with structurally similar proteins shows a well-preserved two-domain alpha helical bundle fold (Figure 2). Both structural and sequence alignments, however, show that there are various insertions and deletions that lower the statistical agreement in the comparisons. RMSD values between HblL_1_ and App6Aa2 (formerly Cry6Aa; 5KUC, 5KUD), NheA (4K1P) [18] and HblB (2NRJ) [13] are 4.7 Å, 4.2 Å, 4.1 Å and 1.5 Å, respectively indicating greatest similarity to the HblB protein that is one of its partners in the Hbl tripartite toxin. Some of the long helices in HblL_1_ are discontinuous and a loop (residues 194–201), bordered by paired Gly residues, seen between α3a and α3b (Figure 1b) is reminiscent of a “wing” loop seen further towards the N-terminal end of the equivalent helix in the App6Aa structure [14]. The function of these regions is not known and equivalent loop structures are not seen in HblB, NheA or HlyE.

Domain 2 in the head region also features the only beta hairpin in the structure (Figure 1), which is a typical feature of this class of protein, forming a hydrophobic beta tongue that has been shown in HlyE to undergo extensive structural rearrangement to contribute to the pore [17]. Mutation in this region of HlyE and App6Aa2, resulted in loss of activity [14,18]. In common with the tongue in HlyE, the HblL_1_ tongue is flanked by glycine residues, which are thought to facilitate a hinge movement of the tongue of HlyE on insertion into the lipid membrane [17]. In HblL_1_, however, this hydrophobic region is more extensive than in the other Hbl proteins, is rich in glycine and alanine residues and contains a central proline (Pro268) residue. The TMHMM (http://www.cbs.dtu.dk/services/TMHMM/, accessed on 31 March 2021) predicts two potential transmembrane (Tm) regions (residues 239–261 and 268–290 in this extended hydrophobic region, Figure S2), compared to only one for HblB (residues V240–A259). It is interesting to note that the third component of the toxin, HblL_2_ has no predicted Tm sequences (other than the putative signal peptide that is present in all of these proteins but removed on secretion). This same distribution of putative Tm sequences also appears in the other *B. cereus* tripartite toxin, Nhe, where NheA has no predicted Tm regions, NheB has two and NheC has one, which may indicate functionally equivalent pairs between the two toxins. This is borne out by investigations that showed initial steps of binding to eukaryotic membranes by the HblB or NheC components (one Tm region) followed by HblL_1_/NheB (two Tm regions) and finally HblL_2_/NheA (no Tm regions) [4].

The App1Ca and App3Aa (formerly known as YaxA/YaxB), two part α‑PFTs from *Yersinia* species (and equivalent proteins from other Gram negative bacteria), are a somewhat distinct group of α‑PFTs. In these proteins, App1Ca is predicted to have a Tm domain that corresponds to the hydrophobic region believed to partially insert in the membrane, while the longer App3Aa region that appears to insert completely through the membrane, is an amphipathic helix [19] (not predicted to be Tm using TMHMM). As a result, the lack of predicted membrane spanning regions in HblL_2_ and NheA does not preclude membrane insertion by these proteins.

### 2.3. Modelling HblL_1_-HblB Interactions

Surface plasmon resonance experiments and enzyme immune assays have indicated the capacity of HblL_1_ and HblB to interact in solution [20]. HblB has also been shown to form high molecular weight complexes in solution and in the presence of HblL_1_, the latter protein can also be incorporated into the complexes [21]. In addition, NheB and NheC, which we propose to be the functional equivalents of HblL_1_ and HblB, have been shown to associate in solution [22,23,24]. We have, therefore, attempted to model how these Hbl components might associate. We used a combined ClusPro and RosettaDock approach to model the complex [25]. ClusPro docking produced 30 clusters and the central models of each were used in local docking refinements using RosettaDock to produce 1000 models for each. All 30,000 models were pooled and ranked according to their total energy value. The 10 lowest scoring models, r1–r10 were then identified (as the thermodynamic hypothesis states that the native structure is located at the global energy minimum and hence, models with the lowest total energy scores are most likely to represent that of the native structure [26]). Of these models, total energy ranged from −1233.8 to −1235.6 Rosetta Energy Units (REU) (Table 2). To analyse the interfacial interactions, PDBePISA was utilised [27]. Interface area ranged from 966 to 1042.3 Å^2^ and all models were found to contain a number of interfacial hydrogen bonds and salt bridges (Table 2). In addition, solvation free energy gain (Δ^i^G) ranged from −1.1 to −5.8 kcal mol^−1^.

**Figure 3:**
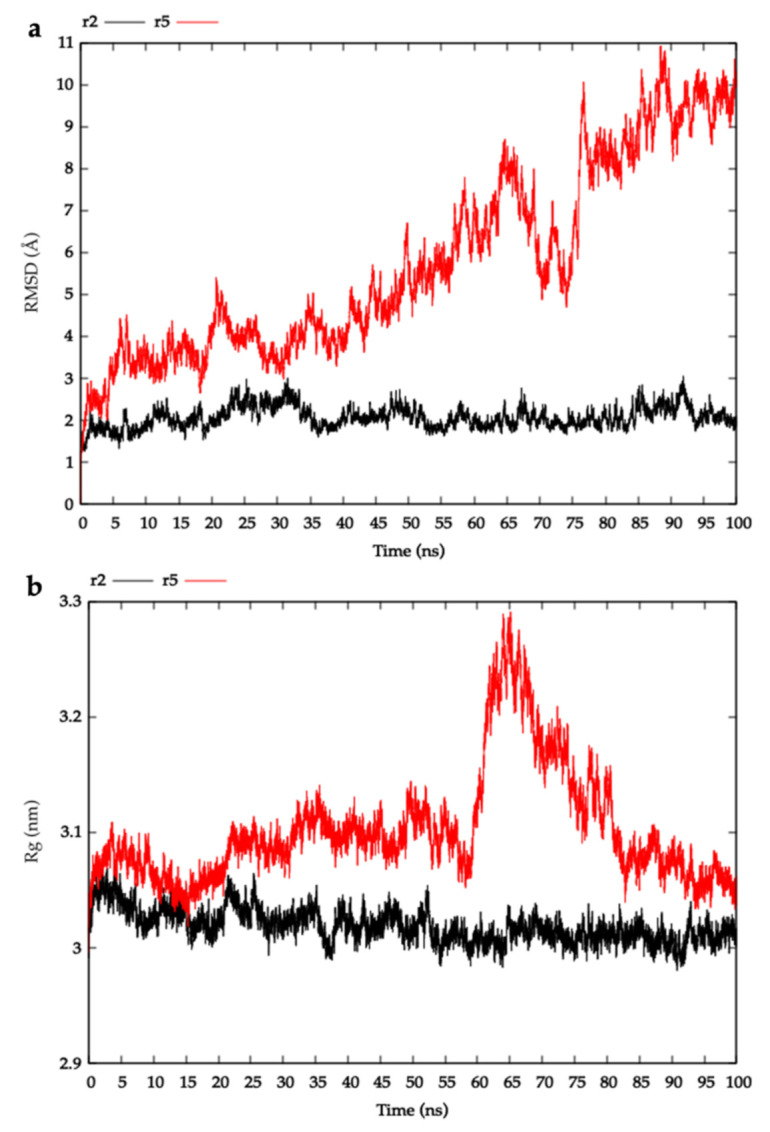
Molecular dynamic analysis of HblL_1_-HblB complexes. (**a**) Root-mean-square deviation of the position of backbone atoms in HblL_1_-HblB modelled complexes throughout 100 ns MD simulations. (**b**) Radius of gyration (R_g_) of HblL_1_-HblB modelled complexes throughout 100 ns MD simulations. Plots for the most (model r2) and least (model r5) stable complexes are shown for comparison (plots for all complexes can be viewed in Figure S3).

To assess the structural stability of the lowest scoring HblB–HblL_1_ models generated, 100 ns molecular dynamics (MD) simulations were performed and the root-mean-square deviation (RMSD) of the position of backbone atoms was calculated as a function of time. In simulations where models are unstable and drift away from their starting structures, a large RMSD value is expected. On the other hand, where models are stable and remain close to their starting structures, a low RMSD value is expected. Out of models r1–r10, model r2 (Figure 3a) was found to exhibit the lowest RMSD values, moving no more than 2.5 Å away from its starting structure. This result suggests that model r2 remains in a stable configuration throughout the 100 ns simulations performed. In the past, MD simulations and subsequent RMSD calculations have been utilised to identify a model with the structure that was closest to the experimentally derived structure for a protein complex (G3:HER2_IV) [28], indicating the ability of MD simulations to discriminate near-native structures from non-native structures. In addition to RMSD, the radius of gyration (R_g_), which indicates the compactness of the overall protein structure, was calculated as a function of time (Figure 3b). Large increases in R_g_ are associated with a looser packing of the model and hence may be used to indicate structural stability [29]. Figure 3b shows that the R_g_ of model r2 remained relatively constant throughout 100 ns simulations, indicating good structural stability. On the basis of our MD results, model r2 may represent a possible structure of an HblB-HblL_1_ complex.

**Figure 4:**
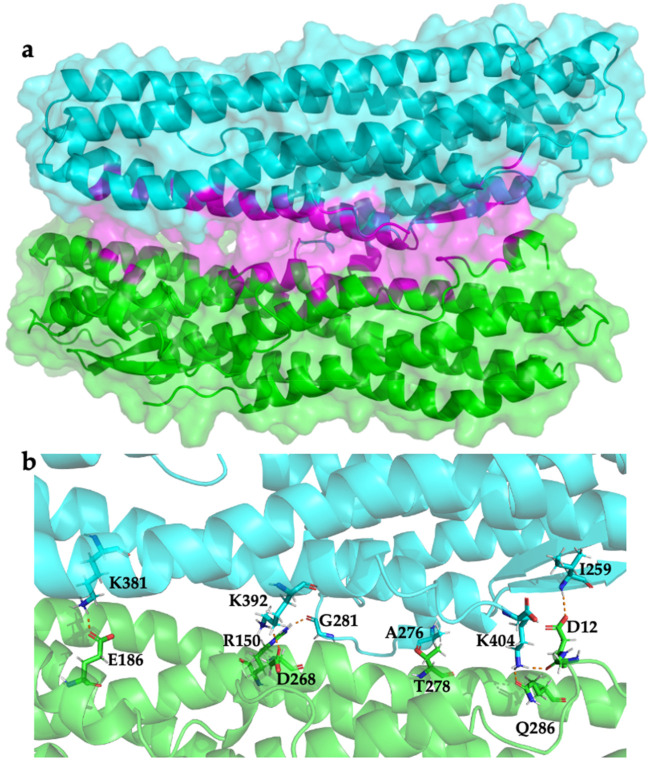
HblL_1_-HblB model r2. (**a**) the predicted dimer structure is illustrated showing HblL_1_ (cyan) and HblB (green) with the HblL_1_ beta tongue buried in the interface region (magenta); (**b**) polar interaction network via H-bonds and salt bridges as shown in Table 3.

With respect to the overall structure of each component, r2 represents a head-to-tail dimer (Figure 4) with a number of H bonds and salt bridges in the interface (Table 3). In the computed complex, the beta tongue region of HblL_1_ is buried against the tail region of HblB; the β-tongue of HblB does not play a part in the dimer interaction. It has been shown that the Hbl structural homolog HlyE, forms head to tail dimers that bury hydrophobic patches on crystal formation although SEC shows it to be monomeric in solution [18]. The HlyE variants from different *E. coli* strains show a differential propensity for dimer formation [30] and dimers can be detected during HlyE pore assembly [16]. We can speculate that an association of HblL_1_ with HblB may have a role in assisting the solubility of HblL_1_ by burying its large, hydrophobic beta tongue (residues 234–293) and an association may also play a role in priming of pore formation by the tripartite toxin. Clearly further analyses will be necessary to confirm these speculations.

### 2.4. The Alpha Helical Pore Forming Toxins

It is interesting to consider the roles of individual components in the alpha helical pore forming toxins. For the Hbl proteins, as with the Nhe toxin, three components are required to elicit activity but we know that structural homologs of these proteins such as HlyE and App6 are able to exert toxicity through the action of single proteins. In this regard, the App6 family proteins show an interesting operon arrangement with App6Aa followed by an inverted repeat sequence and then 3 further CDSs, each predicted to encode proteins in the App/Hbl/Nhe/HlyE structural family (Bt plasmid pBMB0228 accession CP002486—a distinct arrangement of similar CDSs is associated with the App6Ba gene eg accession CP015251). These additional CDSs are not necessary for App6Aa activity (and, although the first CDS has been shown to influence expression of App6Aa [31], no independent toxicity for the products of these CDSs has been demonstrated). Other, more distant structural relatives of the Hbl-like toxins, eg App1Ca and App3Aa (formerly known as YaxA/YaxB) exert their activity as two-part alpha-helical toxins [19]. The evolutionary processes that mediate development of multicomponent and single component toxins in this family and the roles of the various components in receptor binding and pore formation are not yet clear and further structure function investigations will be needed to probe these issues in more detail.

## 3. Conclusions

In this work we have elucidated the structure of the L_1_ component of the Hbl tripartite toxin and a further member of the alpha helical pore forming toxin family. Features of the toxin that may relate to its function are highlighted and features such as predicted transmembrane sequences that may make it the functional equivalent of the NheB component of the Nhe tripartite toxin. We have also modelled a potential complex between our newly solved HblL_1_ protein and the HblB partner protein. Further studies to solve the structure of the final element required for toxicity, the HblL_2_ protein, and complexes between all of the subunits—both in solution and in their membrane interacting states, will be required to extend this work and to understand the mechanism of this toxin fully.

## 4. Materials and Methods

### 4.1. Protein Purification

HblL_1_ protein (accession EEM59260.1, with C‑terminal His tag) was expressed in *Bacillus anthracis* strain BH460 and purified using the C‑terminal His tag as previously described [4] and stored frozen in 10 mM HEPES, 0.5 mM EDTA, pH 7.5 until use.

### 4.2. Crystallisation and Structure Determination

Crystallisation trials were conducted using the PACT HT96 screen (Molecular Dimensions) and good quality crystals were formed in 0.1 M cacodylate Bis Tris propionate, 25% *w*/*v* PEG 1500, pH 4.0. One crystal was used to determine the structure (Figure S5). Diffraction data were collected at Beamline I04-1, Diamond Light Source, Harwell, UK (diamond beamline I04-1, DLS visit mx18812-4, 25-10-2018). Data reduction was completed with XIA2, XDS, AIMLESS and TRUNCATE in the CCP4 Package. Merged reflection data were uploaded onto the AMPLE server [32] using models generated by the ROBETTA server [33,34,35,36]. Ample generated a C_α_ backbone model. COOT [37] was used to correct chain IDs and residue numbering manually. Side chains were filled in using the mutate residue range function. The structure was refined using iterative rounds of REFMAC [38] and manual model building with COOT until the structure reached convergence.

### 4.3. Molecular Docking

#### 4.3.1. Docking

Molecular docking was performed using a naïve approach with no presumed docked HblL_1_-HblB interface as outlined in Appendix A. The structure of HblB was obtained by extracting residues 2–341 from the crystal structure (PDB 2NRJ) [13]. Missing residues _200_SD_201_ were modelled using the Rosetta remodel executable [39]. The structure of HblL_1_ was obtained by extracting residues 46–404 from the crystal structure elucidated as part of this work. First, the ClusPro 2.0 server was utilised to perform a global docking search [40]. ClusPro employs a fast Fourier transform (FFT)-based algorithm, which places the receptor protein on a fixed grid and the ligand on a moveable grid. The interaction energies are calculated as a correlation function for each grid point, thus enabling sampling of billions of possible protein-protein conformations. All possible conformations are scored, and the 1000 lowest energy models are subjected to RMSD clustering followed by energy minimisation. The output is based on cluster size, with the largest cluster ranking highest and the smallest cluster ranking lowest. For each cluster, ClusPro outputs the central model, the energy score of this model, and the cluster size. Here, the HblB and HblL_1_ starting structures were provided as the receptor and ligand, respectively. Some 30 clusters were identified.

Further analysis involved carrying forward central models from these clusters for independent local docking using RosettaDock, which allows refinement of the docking approach [41]. This algorithm includes a low-resolution stage during which side chains are represented as pseudo-atoms and rigid body perturbations occur. This is followed by a high-resolution stage during which smaller rigid body perturbations, side-chain optimisation, and energy minimisation occur. Output models are scored using the Rosetta *ref2015* score function [42]. This score function is composed of a number of weighted energy terms that are used to calculate the total energy of models in Rosetta Energy Units (REU). To ensure side chains were present in their lowest energy conformation, each model was prepacked using the docking_prepack_protocol.macosclangrelease executable. For docking, the docking_protocol.macosclangrelease executable was used and to improve side chain modelling, unbound rotamer conformations were provided [42]. For each docking search, 1000 models were generated. The models from all 30 clusters (30,000 models) were pooled and ranked according to their total energy score. Subsequently, the 10 models from this pool with the lowest energy scores were carried forward for structural interface analysis and molecular dynamics (named r1–r10, with r1 being the model with the lowest energy score and r10 being the model with the highest energy score).

#### 4.3.2. Interface Analysis

For interface analysis of models, the PDBePISA (PISA) web server was utilised [27]. PISA enabled calculation of the interface area (Å^2^) and solvation free energy gain (∆^i^G, kcal mol^−1^), as well as the identification of interfacial hydrogen bonds and salt bridges.

#### 4.3.3. Molecular Dynamics

To assess the structural stability of models, molecular dynamics (MD) simulations were performed using GROMACS (v.2020.1) [43] on Cardiff University’s High-Performance Computer, Hawk. Each model was centred in a cubic box, solvated using a 3-point solvent model, and neutralised via the addition of 17 Na^+^ ions. The AMBER99SB force field was used to provide the parameters of atoms, molecules, and their interactions with each other [44]. Solvated protein systems were energy minimised and equilibrated. Energy minimisation was performed using the steepest descent algorithm in step sizes of 0.01 with a maximum number of 50,000 steps. Equilibration was performed in two stages. The first stage was performed using an isothermal-isochoric ensemble to stabilize the temperature of the system. Pressure coupling was not applied. The second stage was performed using an isothermal-isobaric ensemble to stabilize the pressure of the system. Pressure coupling using a Parrinello-Rahman barostat was applied. In both stages, equilibration was carried out for 100 ps.

Next, 100 ns MD simulations were initiated. Simulations were performed at a constant temperature of 300 K, using a velocity rescaling thermostat, separate couplings for proteins and non-proteins, and a relaxation constant of 0.1 ps. To maintain a constant pressure of 1.0 bar, a Parrinello-Rahman barostat with an isothermal compressibility of 4.5 × 10^−5^ bar^−1^ was used. The pressure was coupled isotropically with a coupling constant of 2.0 ps. Newton’s equations of motion were integrated with a timestep of 2 fs. The Particle-Mesh Ewald method and a 10 Å cut off were used to calculate non-bonded long-range electrostatic interactions. Similarly, for van der Waals interactions, a 10 Å cut off value was used. All bonds involving hydrogen atoms were constrained using the LINCS constraint algorithm.

To assess the structural stability of docked models, the RMSD of the position of backbone atoms and R_g_ of models were analysed as a function of simulation time using GROMACS modules gmx rms and gmx gyrate. The Visual Molecular Dynamics (VMD, v.1.9.4) program was utilised to visualise simulations and the Gnuplot (v.5.2) program was employed to produce the graphics associated with this work [45].

## Figures and Tables

**Table 1:** Crystallographic data statistics for HblL_1_.

PDB Entry	HblL_1_
Data Collection	
Accession Code	7NMQ
Wavelength	0.91587
Crystallisation Condition	0.1 M cacodylate Bis Tris propionate, 25% *w*/*v* PEG 1500, pH 4.0
Crystal Data	
*a, b*, *c* (Å)	36.66, 72.96, 133.05
*α, β, γ*	90.0, 90.0, 90.0
Space group	P 2_1_ 2_1_ 2_1_
Resolution (Å)	1.36–63.97
Outer shell	1.36–1.38
*R*-merge (%)	6.3 (135.7)
*R*-pim	3.9 (95.2)
*R*-meas (%)	7.0 (153.4)
CC1/2	0.999 (0.397)
I/σ(I)	12.5 (1.0)
Completeness (%)	99.3 (99.0)
Multiplicity	5.5 (4.6)
Total Measurements	423,755 (16,876)
Unique Reflections	77,106 (3698)
Wilson B-factor(Å^2^)	12.7
Refinement Statistics	
Non-H Atoms	3498
R-work reflections	73,264
R-free reflections	3764
R-work/R-free	16.2/19.9
Rms Deviations	
Bond lengths (Å)	0.013
Bond Angles (°)	1.686
^1^ Coordinate error	0.048
Mean B value (Å^2^)	17.4
Ramachandran Statistics	
Favoured/allowed/Outliers	290/7/0
%	97.4/2.6/0.0

Figures in brackets refer to outer resolution shell, where applicable. ^1^ Coordinate Estimated Standard Uncertainty in (Å), calculated based on maximum likelihood statistics.

**Table 2:** Computed properties of HblL_1_-HblB modelled complexes.

	RosettaDock		PDBePISA			
Model	Total Energy (REU)	Interface Score (REU)	Interface Area (Å^2^)	Δ^i^G (kcal mol^−1^)	H Bonds	Salt Bridges
r1	−1235.6	−30.3	1042.3	−2.3	6	4
r2	−1235.5	−32.8	1000.9	−3.9	7	2
r3	−1234.9	−30.0	968.1	−3.2	4	2
r4	−1234.8	−32.1	1016.2	−3.0	7	3
r5	−1234.7	−30.9	1025.9	−3.8	6	4
r6	−1234.6	−31.0	1034.9	−3.7	6	4
r7	−1234.3	−30.6	1021.6	−3.0	6	5
r8	−1234.3	−29.3	1004.6	−5.8	4	3
r9	−1234.0	−30.4	966.0	−1.1	8	2
r10	−1233.8	−30.2	991.3	−3.9	7	4

**Table 3:** H bond and salt bridge interactions in model M1r2.

HblL_1_	HblB	Type of Interaction
Ile259	Asp12	Hydrogen bond
Ala276	Thr278	Hydrogen bond
Gly281	Arg150	Hydrogen bond
Lys381	Glu186	Hydrogen bond, salt bridge
Lys392	Asp268	Hydrogen bond, salt bridge
Lys404	Gln286	Hydrogen bond
Lys404	Asp12	Hydrogen bond

**Figure A1:**
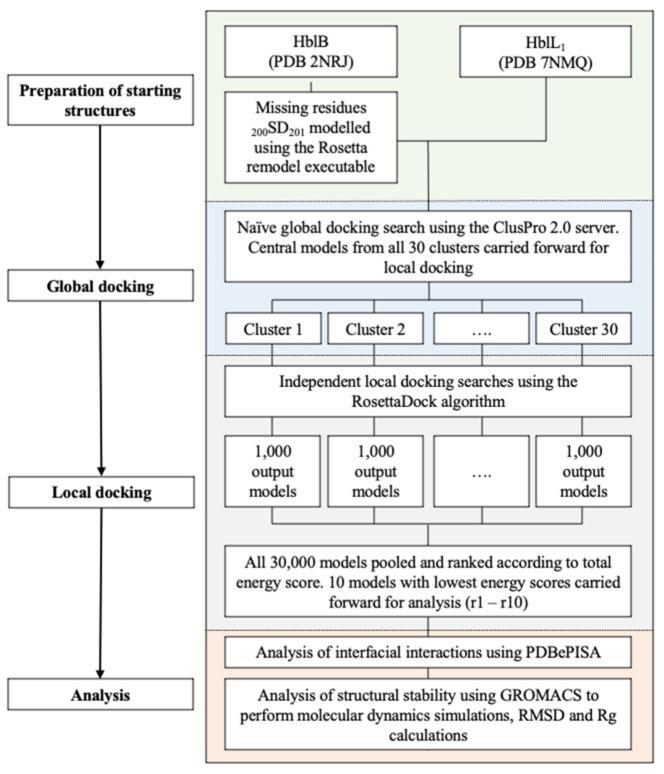
Flow diagram illustrating the modelling steps in predicting HblL_1_-HblB interactions.

## Data Availability

The data presented in this study are openly available in the Protein Data Bank at www.rcsb.org (accessed on 30 March 2021) with accession code 7NMQ.

## Supplementary Materials

The following are available online at https://www.mdpi.com/article/10.3390/toxins13040253/s1, Figure S1: Crystal packing of HblL1. Figure S2: Predicted transmembrane regions in HblL1. Tm region 1, residues 239–261 is shown in yellow; Tm region 2, residues 268–290 is shown in light brown. Figure S3: Molecular dynamic analysis of HblL1-HblB complexes. (a) Root-mean-square deviation of the position of backbone atoms in HblL1-HblB modelled complexes r1–r10 throughout 100 ns MD simulations; (b) Radius of gyration of HblL1-HblB modelled complexes r1–r10. Figure S4: HblL1 crystal and diffraction image. (a) single crystal from which data were collected; (b) single frame from diffraction pattern derived from this crystal.
