# Supplementary material for: The Crystal Structure of Bacillus cereus HblL1
Source: Toxins (Basel). 2021 Mar 31;13(4):253. doi: 10.3390/toxins13040253 (PMC8065917; doi:10.3390/toxins13040253)
Supplement: Supplementary file 1 [file toxins-13-00253-s001.pdf]

Supplementary Materials: The Crystal Structure of *Bacillus cereus* HblL1

Harley L. Worthy, Lainey Williamson, Husam Sabah Auhim, Stephen H. Leppla, Inka Sastalla, D. Dafydd Jones, Pierre J. Rizkallah and Colin Berry

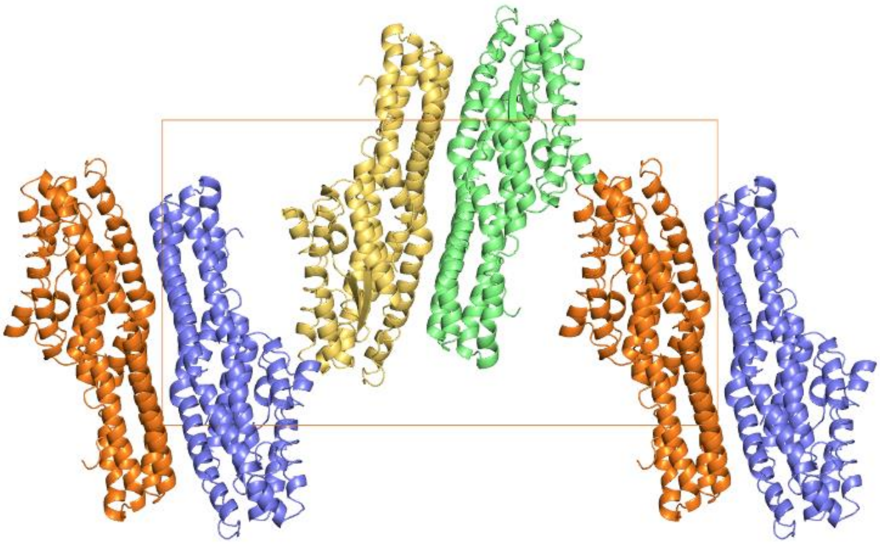

Figure S1. Crystal packing of HblL1.

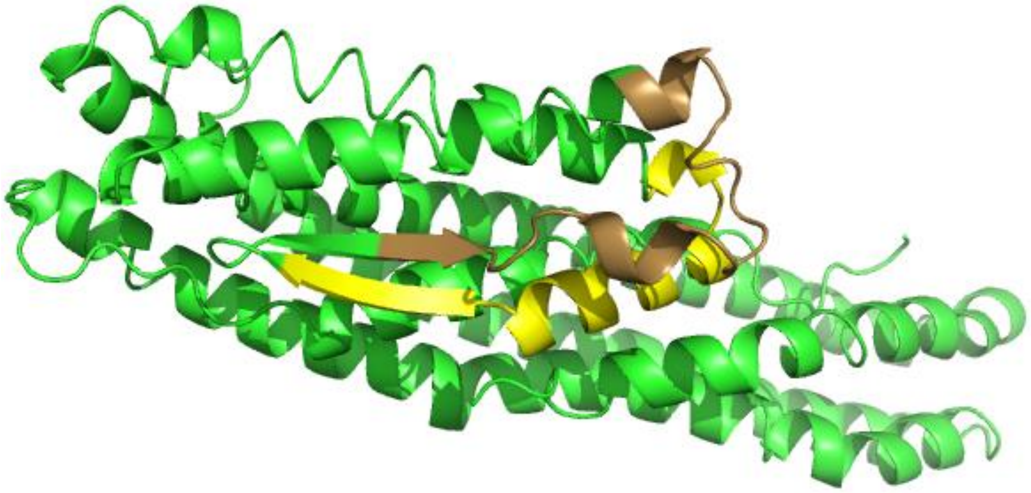

Figure S2. Predicted transmembrane regions in HblL1. Tm region 1, residues 239-261 is shown in yellow; Tm region 2, residues 268-290 is shown in light brown.

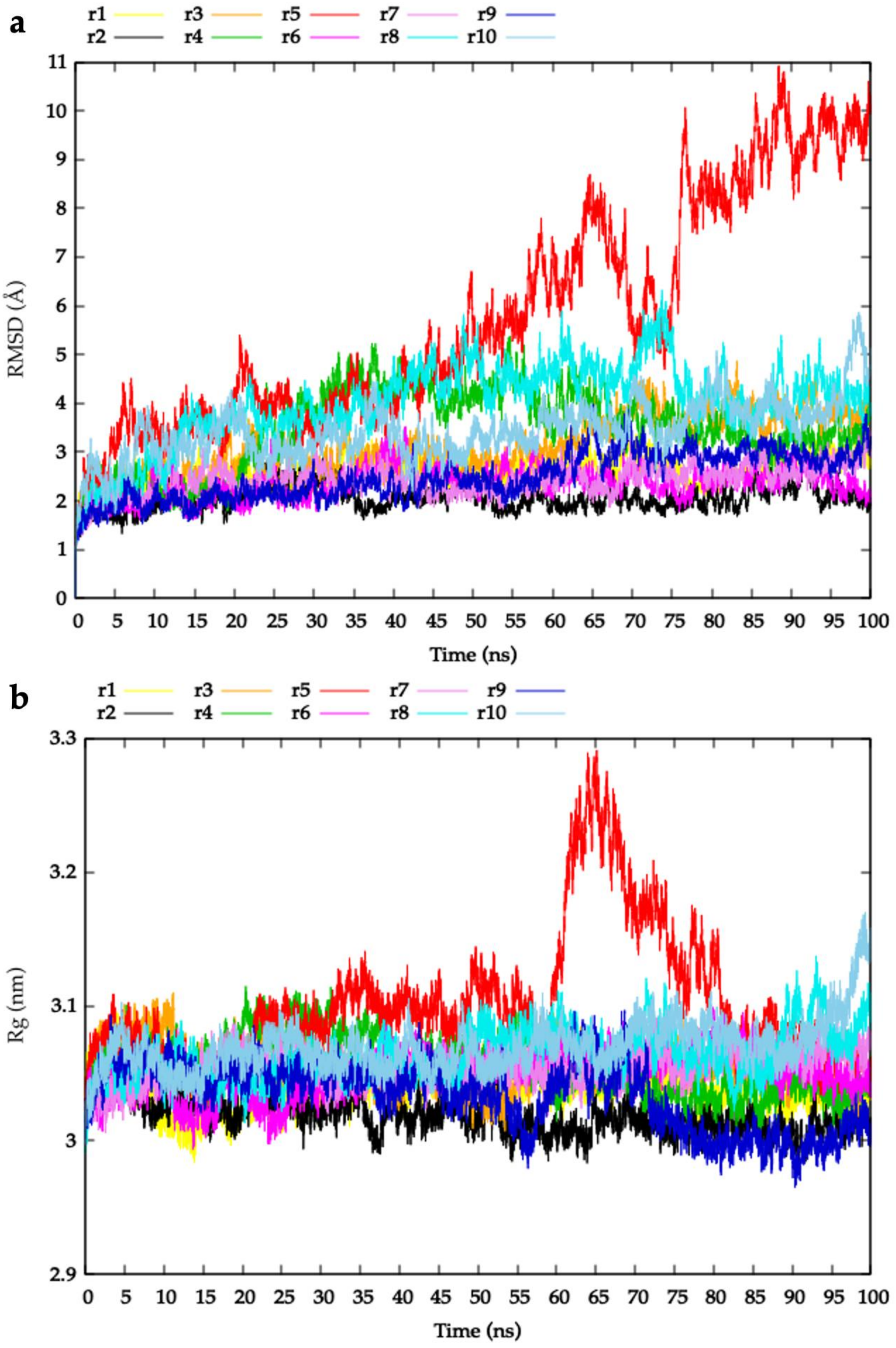

Figure S3. Molecular dynamic analysis of HblL$_1$-HblB complexes. (**a**) Root-mean-square deviation of the position of backbone atoms in HblL$_1$-HblB modelled complexes r1 - r10 throughout 100 ns MD simulations; (**b**) Radius of gyration of HblL$_1$-HblB modelled complexes r1 - r10.

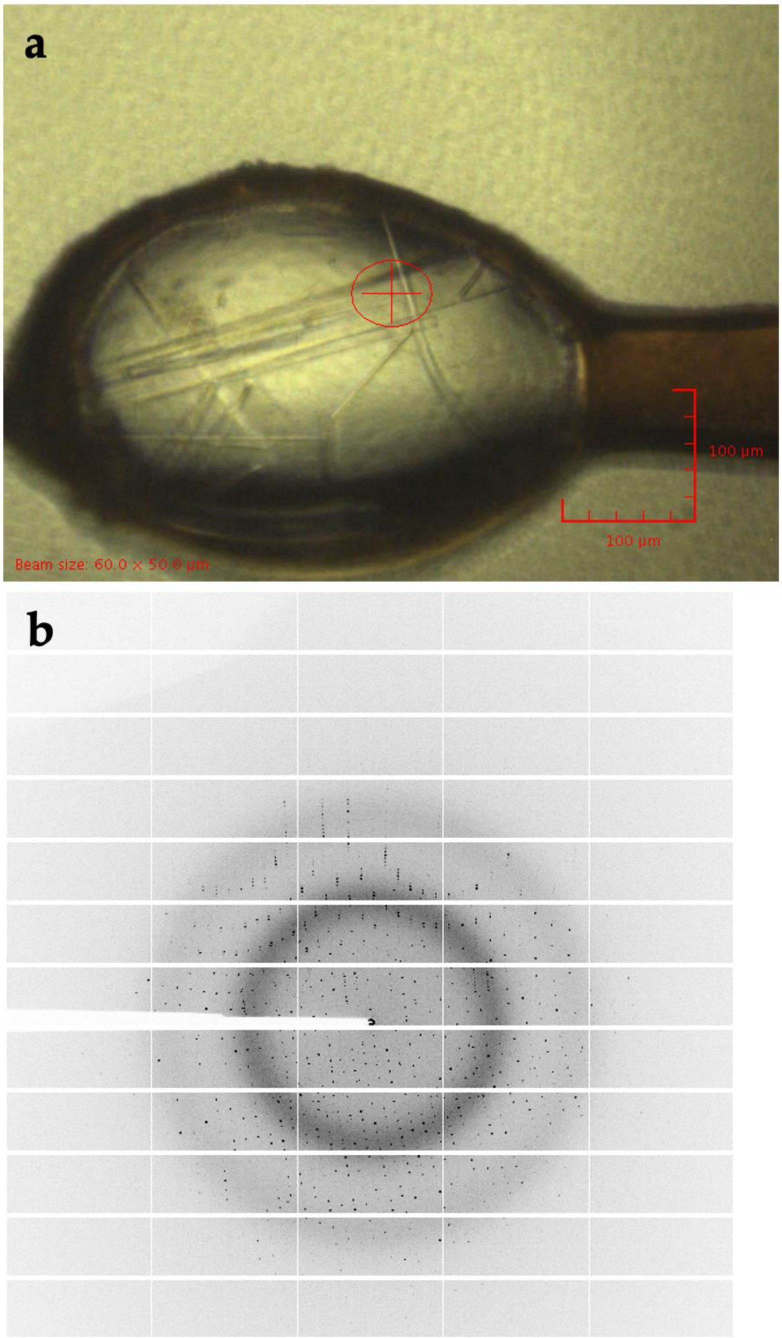

Figure S4. HblL1 crystal and diffraction image. (**a**) single crystal from which data were collected; (**b**) single frame from diffraction pattern derived from this crystal.
